# Electrical stimulation influences chronic intermittent hypoxia-hypercapnia induction of muscle fibre transformation by regulating the microRNA/Sox6 pathway

**DOI:** 10.1038/srep26415

**Published:** 2016-05-20

**Authors:** Shiyuan Huang, Lu Jin, Jie Shen, Ping Shang, Xianxun Jiang, Xiaotong Wang

**Affiliations:** 1 The Centre of Neurology and Rehabilitation, the Second Affiliated Hospital of Wenzhou Medical University, Wenzhou, China

## Abstract

Chronic obstructive pulmonary disease can cause muscle fibre transformation due to chronic intermittent hypoxia-hypercapnia (CIHH). Studies have shown that high expression of Sox6 in muscle could suppress type-I fibres through downregulating the PPARβ (peroxisome proliferator-activated receptor β)/ERRγ (oestrogen-related receptor γ)/microRNA pathway. However, whether this pathway is involved in CIHH-induced muscle fibre transformation is unknown. Electrical stimulation (ES) is an effective approach to ameliorate muscle dysfunction. Here, we explored the effects of ES on CIHH-induced muscle fibre transformation and the microRNA/Sox6 pathway. After CIHH exposure, both the soleus (SOL) and gastrocnemius (GC) muscles showed decreased type-I fibres. The PPARβ/ERRγ/mir-499&208b (PEM, for GC) and PPARβ/mir-499&208b (PM, for SOL) signalling cascades were suppressed, followed by elevated Sox6 expression. Low frequency electrical stimulation (LFES) activated the PEM/PM pathway and enhanced type-I fibre numbers through suppressing Sox6 in SOL and GC. High frequency electrical stimulation (HFES) promoted type-I fibre expression through activating the PEM pathway in GC. Although PPARβ expression and type-I fibres were suppressed in SOL after HFES, no significant change was found in mir-499&208b/Sox6 expression. These results suggest that the microRNA/Sox6 pathway is disturbed after CIHH. Both low and high frequency electrical stimulations induce muscle fibre transformation partly through regulating the microRNA/Sox6 pathway.

Chronic obstructive pulmonary disease (COPD) is the fourth leading cause of death in humans^1^. The high mortality of this disease is closely related to skeletal muscle dysfunction^2^. The fact that pulmonary transplantation cannot fully retrieve exercise capacity indicates that skeletal muscle dysfunction may be an independent aetiology leading to low exercise performance^3^. Type-I fibre reduction is one of the primary disorders and is closely associated with low muscle endurance in COPD patients^4^. However, the mechanism regulating this process is still obscure. In this study, we used rats with chronic intermittent hypoxia-hypercapnia (CIHH) to mimic the pathological process of COPD and investigate the potential mechanism underlying the transformation of skeletal muscle fibre type. Previously, our CIHH rat model successfully mimicked the pathological process of COPD, impairing rat cognitive function^5^.

Myosin heavy chains (MyHCs) are important structures for muscle contraction. Because there are different isoforms of MyHCs, muscle fibres can be categorized as type-I and type-II. Type-I fibres are mitochondrial-abundant and resistant to fatigue (e.g., SOL)^6^. Type-II can be further divided into IIa, IIb and IIx sub-groups, which have a higher velocity of shortening than type-I fibres. Type-II fibres, especially type-IIb, also named glycolytic fibres, are very sensitive to fatigue (e.g., EDL). Type-IIa and type-IIx are also known as fast-twitch oxidative glycolytic fibres, which have a faster twitch speed and lower levels of oxidative enzymes than type-I fibres (e.g., GC). Muscle fibre transformation can be initiated by postnatal external stimuli: a slow to fast switch can be induced by disuse^7^ or hypoxia^8^, while a fast to slow switch can be induced by endurance training or low frequency electrical stimulation, for example^9^,^10^. Exercise endurance impairment occurs frequently in COPD patients and is mainly attributed to the fibre transformation from type-I to type-II^11^.

MicroRNAs are small (19–22 nt) noncoding RNAs that play an important role in diverse physiological regulation processes through repressing gene expression at the posttranscriptional 3-untranslated region (UTR)^12^. Mir-499 and mir-208b are muscle-specific microRNAs specifically expressed in muscles. A previous study confirmed that mir-499 and mir-208b overexpression induces the upregulation of type-I fibre numbers, partly through suppressing Sox6^13^, a member of the Sox (Sry-related high motility group) family and a powerful slow fibre repressor, which may function as an architectural protein in transcription factor activation^14^. The nuclear receptor ERRγ (oestrogen-related receptor γ) is effective in the regulation of muscle mitochondrial function, muscle fibre type and revascularization^15^. A recent study revealed that both Myh7 (which encodes mir-208b) and Myh7b (which encodes mir-499) had putative binding sites with ERRγ, and overexpression of ERRγ would lead to upregulation of mir-499 and mir-208b^16^. These results provided reliable evidence that mir-499 and mir-208b could be regulated by ERRγ. Meanwhile, the researchers found that overexpression of PPARβ (peroxisome proliferator-activated receptor β) could also significantly increase mir-499 and mir-208b expression, partly through activating ERRγ. A study in COPD patients showed that muscle PPARβ was significantly downregulated and was correlated with impaired muscle oxidative capacity^17^. Another study, focused on establishing the relationship between microRNA levels and exercise performance among COPD patients, found that decreased exercise tolerance was correlated with the downregulation of muscle mir-499^18^. These clues lead us to conjecture that the PPARβ/ERRγ/microRNA/Sox6 axis might be disturbed and is related to the slow fibre reduction caused by CIHH.

Electrical stimulation is a valid technique that can improve the muscle metabolic state^19^, endurance and power. Different electrical stimulation frequencies have different effects on muscle fibre type. Generally, LFES can induce type-I fibre expression and elevate muscle endurance^20^, while HFES can increase muscle strength^21^. Here, we sought to explore the effects of electrical stimulation on type-I fibres across two different frequencies, with a specific focus on the PPARβ/ERRγ/microRNAs/Sox6 axis in CIHH rats.

## Results

### Pathological changes after electrical stimulation in SOL

**Figure 1:**
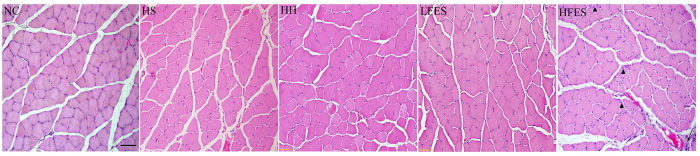
H & E staining of SOL. These pictures show the pathological events in the SOL in each group. The scale bar represents 50 μm. The arrows indicate inflammatory cell infiltration. The triangles indicate internal nuclei. NC: normal control group; HS: hypoxia-hypercapnia+ sham stimulation group; HH: hypoxia-hypercapnia group; LFES: hypoxia-hypercapnia + low frequency electrical stimulation group; HFES: hypoxia-hypercapnia + high frequency electrical stimulation group.

Hematoxylin-eosin (H & E) staining revealed (Fig. 1) many more inflammatory cells in the HFES group compared to the NC, HS, HH, and LFES groups. Furthermore, in the HFES group, we found internal nuclei in some fibres, and the fibrous septum was infiltrated with more inflammatory cells.

### Increased numbers of type-I fibres in SOL after low frequency electrical stimulation

**Figure 2:**
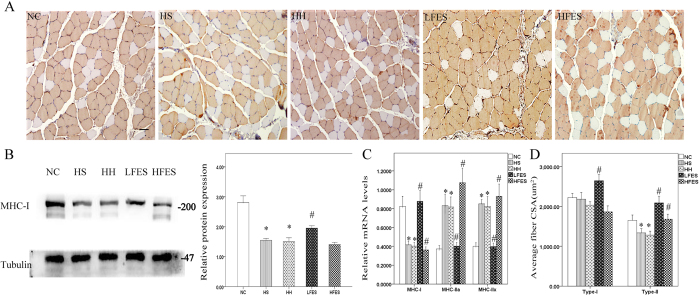
Fibre type change of each group in SOL. (**A**) Immunochemical staining for type-I fibres in SOL. Scale bar = 50 μm; (**B**) Western blotting for MHC-I. The optical density values are normalised to their respective tubulin loading control, and the means ± SD are graphed (relative expression) to semi-quantitatively compare the protein levels; (**C**) Quantification of MHC-I, IIa, and IIx gene expression in SOL; (**D**) Average cross-sectional area (CSA) of type-I and type-II muscle fibres in SOL; *P < *0.05* vs. the NC group; ^*#*^*P* < *0.05* vs. the HS or HH group. NC: normal control group; HS: hypoxia-hypercapnia+ sham stimulation group; HH: hypoxia-hypercapnia group; LFES: hypoxia-hypercapnia+ low frequency electrical stimulation group; HFES: hypoxia-hypercapnia+ high frequency electrical stimulation group.

To explore the effect of electrical stimulation on SOL fibre type transformation, we applied immunochemistry to visualize the type-I fibres. The results showed (Fig. 2A) that the HS and HH groups had significantly fewer type-I fibres compared to the NC group. After a two-week low frequency electrical stimulation training, SOL type-I fibres were significantly increased compared to the HS or HH group. However, under high frequency electrical stimulation, SOL showed no significant change in type-I fibres. To further validate our results, we used western blotting and qRT-PCR (Fig. 2B,C). Western blotting corroborated the immunochemistry results. The qRT-PCR showed decreased MHC-I and increased MHC-IIa and MHC-IIx gene expression levels in the HS and HH groups compared to the NC group. In addition, significantly increased MHC-I and decreased MHC-IIa/IIx mRNA was observed in the LFES group compared to the HS and HH groups. Finally, the MHC-I mRNA level was obviously downregulated. On the other hand, MHC-IIa and MHC-IIx gene expression levels were significantly increased in the HFES group compared to the HS and HH groups. We also quantified the cross-sectional area (CSA) of type-I and type-II fibres in each group (Fig. 2D). After CIHH exposure, we found no significant change in CSA in type-I fibres compared to the NC group. However, there was a discrepancy between the LFES and HFES groups because LFES elevated the type-I and type-II fibre CSA, but HFES only increased the type-II fibre CSA.

### Electrical stimulation activates the microRNA/Sox6 pathway in SOL

**Figure 3:**
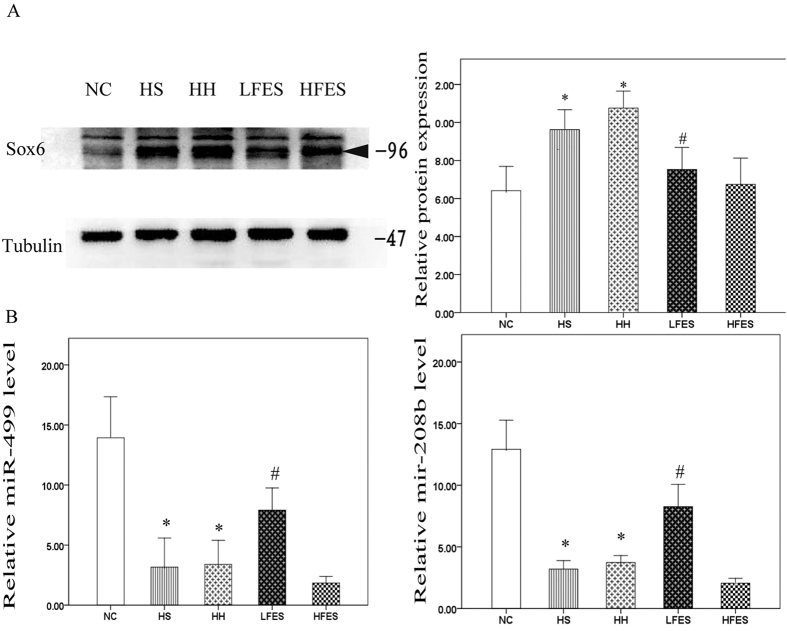
Electrical stimulation activated the microRNA/Sox6 pathway in SOL. (**A**) Western blotting analysis of Sox6 expression. Tubulin was used as the loading control. The triangle shows the target band. (**B**) qRT-PCR analysis of mir-499 and mir-208b. Values are expressed as the mean ± SEM, *P < *0.05* vs. the NC group, ^*#*^*P* < *0.05* vs. the HS or HH group. NC: normal control group; HS: hypoxia-hypercapnia + sham stimulation group; HH: hypoxia-hypercapnia group; LFES: hypoxia-hypercapnia+ low frequency electrical stimulation group; HFES: hypoxia-hypercapnia+ high frequency electrical stimulation group.

Next, we analysed the signalling pathway that regulates the muscle fibre type transformation. Sox6 is a powerful type-I fibre-suppressing protein. The results showed that in the normal SOL, Sox6 expression was very low (Fig. 3A). However, a four-week chronic intermittent hypoxia-hypercapnia exposure significantly increased Sox6 expression level in the HS and HH groups compared to the NC group. In contrast, low frequency electrical stimulation resulted in a significant reduction in Sox6 expression compared to the HS and HH groups. However, there was no significant change in Sox6 level after high frequency electrical stimulation compared to the HS or HH group. Mir-499 and mir-208b could cooperatively regulate Sox6 expression in skeletal muscle, and their levels were correlated with muscle fibre composition. As shown in our findings (Fig. 3B), four weeks of chronic intermittent hypoxia-hypercapnia significantly reduced both mir-499 and mir-208b expression in the HS and HH groups compared to the NC group, but levels were obviously elevated after low frequency electrical stimulation. Similarly to Sox6, there was no significant change in mir-499 and mir-208b levels in the HFES group compared to the HH and HS groups.

### Low frequency electrical stimulation promotes PPARβ expression in SOL

**Figure 4:**
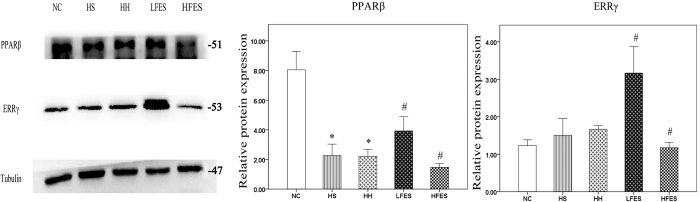
Low electrical stimulation promotes PPARβ expression in SOL. Western blotting was used to establish the expression of PPARβ and ERRγ. The values are normalised to their respective tubulin loading control, and the means ± SD are calculated to compare the protein levels. *P < *0.05* vs. the NC group, ^*#*^*P* < *0.05* vs. the HS or HH group. NC: normal control group; HS: hypoxia-hypercapnia+ sham stimulation group; HH: hypoxia-hypercapnia group; LFES: hypoxia-hypercapnia +low frequency electrical stimulation group; HFES: hypoxia-hypercapnia+ high frequency electrical stimulation group.

We used western blotting to examine the protein levels of PPARβ and ERRγ. In our results (Fig. 4), PPARβ was enriched in normal SOL. After chronic intermittent hypoxia-hypercapnia exposure, PPARβ was lower in the HS and HH groups but was higher after low frequency electrical stimulation. On the contrary, PPARβ was significantly lower after HFES administration. There was no difference in ERRγ expression among the NC, HS and HH groups. However, the ERRγ protein level was significantly elevated after low frequency electrical stimulation administration and was significantly downregulated after high frequency electrical stimulation compared to the HS or HH group.

### Pathological changes after electrical stimulation in GC

**Figure 5:**
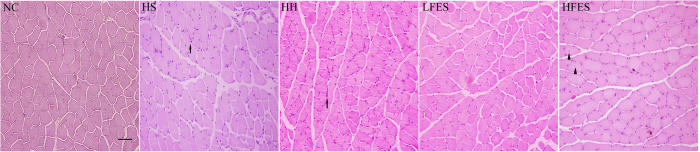
H & E staining of GC. These pictures show the pathological characteristics of GC in each group. Scale bar = 50 μm. The arrows indicate the inflammatory cells. The triangles indicate internal nuclei. NC: the normal control group; HS: the hypoxia-hypercapnia + sham stimulation group; HH: the hypoxia-hypercapnia group; LFES: hypoxia-hypercapnia + low frequency electrical stimulation group; HFES: the hypoxia-hypercapnia+ high frequency electrical stimulation group.

We used H&E staining to investigate the pathological difference among different groups (Fig. 5). In the HS and HH groups, atrophied fibres and inflammatory cells could be found in some regions. Atrophied fibres were also found in the LFES group, but there were almost no inflammatory cells in this group. After high frequency electrical stimulation, we found many hypertrophied fibres in GC. In addition, some fibres with internal nuclei were found in this group.

### Increased type-I fibre in GC after electrical stimulation

**Figure 6:**
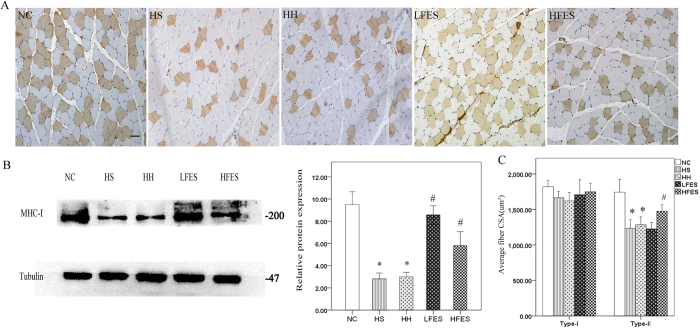
Type-I fibres in each group. (**A**) Immunochemistry staining for type-I fibre in GC. Scale bar = 50 μm. (**B**) Representative Western blotting for MHC-I; the optical density values are normalised to their respective tubulin loading control and the means ± SD are graphed (relative expression) to semi-quantitatively compare the protein levels. (**C**) Average fibre cross-sectional area of type-I and type-II fibres. *P < *0.05* vs. the NC group; ^*#*^*P* < *0.05* vs. the HS or HH group. NC: normal control group; HS: hypoxia-hypercapnia + sham stimulation group; HH: hypoxia-hypercapnia group; LFES: hypoxia-hypercapnia+ low frequency electrical stimulation group; HFES: hypoxia-hypercapnia+ high frequency electrical stimulation group.

Immunochemistry (Fig. 6A) revealed that type-I fibres were decreased after chronic intermittent hypoxia-hypercapnia exposure in the HS and HH groups compared to the NC group. Remarkably, low frequency electrical stimulation significantly increased the number of type-I fibres compared to the HS and HH groups. Unlike SOL, high frequency electrical stimulation also significantly elevated the type-I fibre proportion in GC compared to the HS and HH groups. To confirm this result, we applied western blotting (Fig. 6B). The results paralleled the immunochemistry results. We also studied the type-I and type-II cross-sectional areas among these groups. We found that there were no significant differences in type-I fibre CSA between groups (Fig. 6C). However, after CIHH exposure, type-II fibre CSA was significantly decreased in the HS and HH group compared to the NC group. After low frequency electrical stimulation, we did not find significant change in type-II fibre CSA, but it was obviously increased after high frequency electrical stimulation.

### Electrical stimulation reduces Sox6 expression in GC accompanied by an increase in type-I fibres

**Figure 7:**
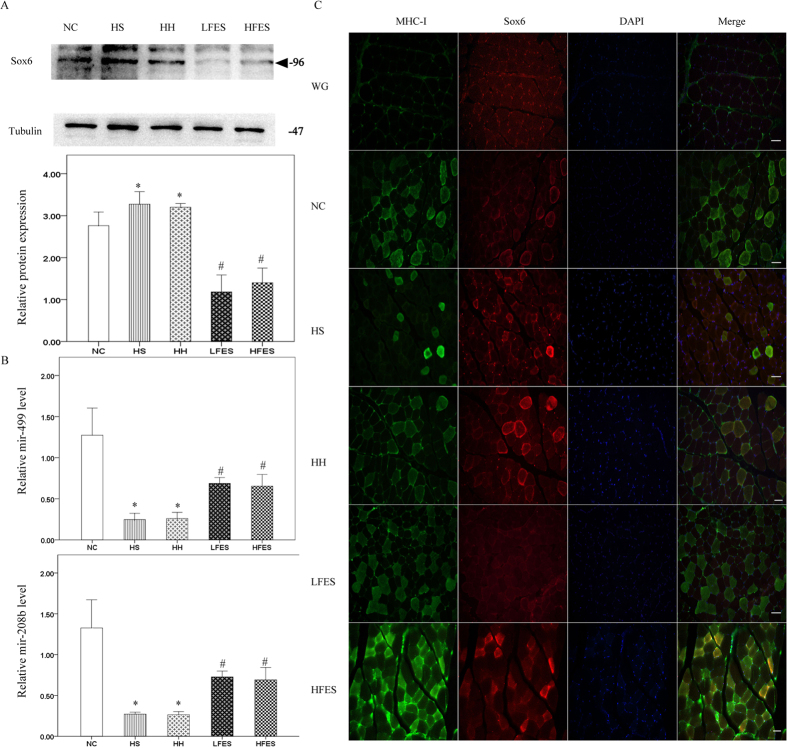
Both low and high electrical stimulation reduce Sox6 expression and induce mir-499 and mir-208b expression in GC. (**A**) Western blotting analysis of Sox6 expression in GC. Tubulin was used as a loading control. (**B**) qRT-PCR analysis of mir-499 and mir-208b. (**C**) Cross-section of gastrocnemius stained for Sox6 and MHC-I by immunofluorescence. Green = MHC-I; Red = Sox6; Blue = DAPI. Scale bar = 50 μm. WG: white GC; *P < 0.05 vs. the NC group, ^*#*^*P* < 0.05 vs. HS or the HH group;. All values represent the mean ± SEM. NC: normal control group; HS: hypoxia-hypercapnia+ sham stimulation group; HH: hypoxia-hypercapnia group; LFES: hypoxia-hypercapnia+ low frequency electrical stimulation group; HF: hypoxia-hypercapnia+ high frequency electrical stimulation.

We then examined the changes in Sox6 and its suppressors mir-499 and mir-208b. As Western blotting showed (Fig. 7A), 4 weeks of chronic intermittent hypoxia-hypercapnia was associated with a significantly higher expression of Sox6. Either low frequency or high frequency electrical stimulation substantially repressed CIHH-induced Sox6 elevation in GC. We also used qRT-PCR to determine mir-499 and mir-208b expression (Fig. 7B). Levels of mir-499 and mir-208b were significantly lower in the HS and HH groups compared to the NC group. Low frequency electrical stimulation significantly upregulated mir-499 and mir-208b expression in the GC muscle fibre.

To directly view and support our results, we performed double immunofluorescence to determine the role of Sox6 on type-I fibre expression (Fig. 7C). Consistent with previous studies, Sox6 was highly expressed in white GC (WG) where there were no type-I fibres. After 4 weeks of CIHH exposure, the Sox6 fluorescence signals were much stronger, while the type-I fibres were significantly decreased compared to the NC group. Both low frequency and high frequency electrical stimulation, however, could significantly enhance type-I fibre numbers, accompanied by the significantly reduced Sox6 fluorescence signals.

### Electrical stimulation promotes PPARβ and ERRγ expression in GC from CIHH rats

**Figure 8:**
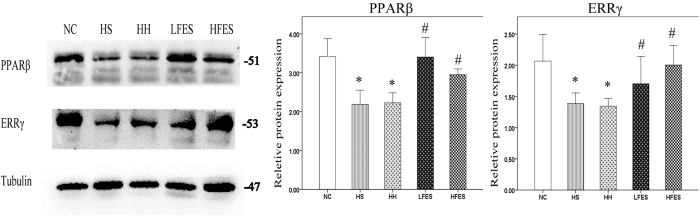
Electrical stimulation promotes PPARβ and ERRγ expression in GC. Western blots for PPARβ and ERRγ are presented. Tubulin was used as a loading control. The values are expressed as the mean ± SEM. *P < 0.05 vs the NC group, ^*#*^*P* < 0.05 vs the HB or the HH group. NC: normal control group; HS: hypoxia-hypercapnia+ sham stimulation group; HH: hypoxia-hypercapnia group; LFES: hypoxia-hypercapnia + low frequency electrical stimulation group. HFES: hypoxia-hypercapnia + high frequency electrical stimulation group.

We next detected PPARβ and ERRγ expression in GC (Fig. 8). PPARβ and ERRγ protein levels were significantly lower in the HS and HH groups compared to the NC group. Low frequency electrical stimulation significantly enhanced PPARβ and ERRγ levels compared to the HS and HH groups. Similar results were found after high frequency electrical stimulation, but the HFES group revealed an even higher ERRγ expression compared to the LFES group.

## Discussion

Reduced muscle endurance is well recognized in COPD patients and is related to exercise limitation^22^. Slow muscle fibre loss can lead to low muscle endurance; however, the mechanism is controversial and not well understood. Previous studies confirmed that Sox6 could reduce slow fibre numbers, but its role in the pathological conditions was less known. Herein, we show that rats under chronic intermittent hypoxia-hypercapnia exposure suffered obviously reduced type-I fibre number in the SOL and GC muscles. However, electrical stimulation can reverse this procedure partly through the derepression of Sox6 by activating the PPARβ/ERRγ/microRNA axis in GC or the PPARβ/microRNA axis in SOL. We also observed that these two muscles react differently toward high frequency electrical stimulation, indicating possibly distinctive fibre type-regulating mechanisms between them.

### CIHH Exposure

After CIHH exposure, we found a significant downregulation in type-I fibres in SOL and GC. A higher percentage of type-IIa and IIx fibres indicates that the fibre transformation procedure was undertaken in both muscles, which was consistent with the findings in COPD patients^11^,^23^. Moreover, fibre cross-sectional area is an important indicator for muscle power^24^. A number of studies have confirmed that hypoxia could contribute to reductions in fibre CSA, and interestingly, fast fibres were more susceptible to hypoxia than slow fibres^25^,^26^. In this study, we observed a significantly reduced CSA of type-II fibres in SOL and GC but no difference in type-I fibres.

Sox6 was enriched in muscles (especially in fast fibres)^27^, and three previous studies had found that Sox6 could act as a slow fibre suppressor during muscle development. These studies were performed in zebra fish (one study)^28^ and mice (two studies)^29^,^30^. However, it was still not clear whether Sox6 could also act as a slow fibre repressor in postnatal periods because the previous studies were based on gene knockout animal models. In this study, we showed a close relationship between Sox6 and type-I fibre expression. As in normal SOL, Sox6 expression was very low. Nevertheless, it was higher in GC. That difference could arise from the greater proportion of type-II fibres in GC compared to SOL. To the best of our knowledge, the relationship between Sox6 expression and hypoxia was only studied in skeletogenesis^31^, which revealed that hypoxia could induce Sox6 expression. Here, we observed that after exposure to chronic intermittent hypoxia-hypercapnia, contrary to the downregulation of MHC-I expression, Sox6 expression was significantly increased in SOL and GC, which might indicate that Sox6 was still functional in muscle fibre type regulation in the postnatal stage.

Mir-499 and mir-208b are highly enriched in slow muscle fibres. Previous scholars found that mir-499 could repress Sox6 expression and augment slow fibre expression in fast-twitch muscle in the zebra fish embryo^32^. A mir-499/208b double knockout (but not knockout separately) could significantly induce MHC-I protein downregulation^13^,^33^. It is noteworthy that Lewis^18^ and Donaldson^34^ showed that COPD patients with a lower percentage of type-I fibres had a trend towards association with lower miR-499 levels. Those findings led us to focus on the synergistic regulation of mir-499/208b in rat muscle fibre type transformation when exposed to CIHH. In our study, we found both mir-499 and mir-208b expression were significantly downregulated in SOL and GC after CIHH exposure.

PPARβ has long been thought to be an important factor in metabolic regulation^35^. PPARβ overexpression mice showed a significant enhancement of running capacity, correlated with an increased number of type-I muscle fibres^36^. How PPARβ exerts its role on skeletal muscle type transition was unknown until ERRγ was found to play a similar role in type-I fibre transition. Zhenji Gan *et al.*^16^ found that PPARβ could act as an upstream regulator to upregulate ERRγ expression. In our rat model, we showed that PPARβ was obviously decreased both in SOL and GC after CIHH exposure, while ERRγ was decreased in GC but unchanged in SOL. The downregulation of PPARβ/ERRγ/microRNA, and the upregulation of Sox6 led us to speculate that the probable suppression of the PEM axis and activation of Sox6 might be partly attributed to the slow fibre reduction after CIHH exposure in GC. Although PPARβ was downregulated, ERRγ in SOL was not significantly changed compared to the NC group. This discrepancy between GC and SOL in ERRγ expression under hypoxia is still unclear. Interestingly, previous research found that neither ERRγ overexpression nor knockout would have an effect on SOL type-I fibre expression. Nevertheless, in GC, type-I fibres were significantly increased when ERRγ was overexpressed but were significantly decreased after ERRγ knockout^15^,^37^. Therefore, we concluded that ERRγ was dispensable in fibre type regulation in SOL and there might be compensatory pathways in the downregulation of mir-499 and mir-208b induced by PPARβ after CIHH exposure. To our knowledge, this is the first study demonstrating that the microRNA/Sox6 pathway is impaired in CIHH rat skeletal muscle with muscle fibre transformation.

### Electrical stimulation

Exercise has been broadly used as an effective way for COPD patients to rehabilitate from skeletal muscle dysfunction^38^. However, some limitations prevent patients from participating in exercise training. For instance, patients with low muscle endurance and heart function may not tolerate even light training programs. It has been found, however, that an electrical stimulation (ES) program could partly mimic the exercise procedure and had a similar partial effect on muscle function. In addition, the ES program was free from those limitations. Therefore, it is suitable for muscle rehabilitation in COPD patients with poor exercise performance.

The prevalence of muscle atrophy is relatively high in COPD patients^39^. In our rat model, we also found numerous atrophied muscle fibres in the SOL and GC after CIHH exposure. After HFES administration, there were many hypertrophied muscle fibres in SOL and GC, which was similar to most previous studies. Interestingly, we also found that the CSA of type-II fibres in SOL and GC were selectively increased after HFES, which might indicate that the HFES program was useful to prevent muscle atrophy. It was noteworthy that after high frequency electrical stimulation, some internal nuclei were found in both SOL and GC. The meaning of the internal nuclei is controversial; some researchers believe it might be correlated with muscle hypertrophy^40^ and others attribute it to neuromuscular disorders^41^. We also observed a higher number of inflammatory cells in the SOL after HFES. From the literature, we assumed that this might partly due to the concentric contractions caused by HFES for the activation of NF-κB pathway^42^.

Our investigation then showed significant type-I fibre upregulation in SOL and RG after LFES. We further evaluated the potential role of the PPARβ/ERRγ/microRNA/Sox6 axis in muscle fibre regulation during this procedure. Because ERRγ might not participate in fibre type regulation in SOL, we discuss them separately. Sox6 was obviously suppressed, while both mir-499 and mir-208b were significantly upregulated in SOL and GC after LFES administration. In GC, PPARβ was significantly increased, accompanied by the increased expression of ERRγ after LFES. In SOL, PPARβ and ERRγ expression was also significantly upregulated. As there is no certain evidence that ERRγ plays a role in fibre type transformation, we presume that there might be other ways for LFES to induce mir-499/208b expression and the subsequent Sox6 suppression in SOL. In summary, we find that LFES can induce mir-499/208b expression and inhibit Sox6 expression and was related to the increase of PPARβ/ERRγ expression and type-I fibres in GC. The mechanism through which PPARβ regulating mir-499/208b expression in SOL after electrical stimulation is remained to be disentangled. To our amazement, there was also a significant increase in type-I fibres in GC after HFES, which contradicts most other data. We do not have a reasonable explanation for this discrepancy. Interestingly, a study conducted by Gondin showed that a 75 Hz neuromuscular electrical stimulation could significantly increase quadriceps muscle type-I fibre numbers; they thought the continuous activation of non-slow fibres might be a probable cause^10^. We then investigated the microRNA/Sox6 regulation cascades and found that PPARβ/ERRγ and mir-499/208b were significantly increased, while Sox6 was repressed in GC. These results indicated that this regulation pathway was activated and might lead to the disinhibition of slow fibre by downregulating Sox6 expression. For SOL, although PPARβ was significantly decreased after the HFES program, mir-499/208b and Sox6 were not significantly changed. The MHC-I protein level showed a trend to downregulation, and the mRNA level was significantly decreased. We suppose that there might be other regulating pathways inducing slow fibre reduction after HFES treatment in SOL. For example, PPARβ downregulation might induce other slow fibre repressor activation, such as Purpβ or SP 3 43,44. Collectively, these findings revealed the potential role of microRNA/Sox6 signalling pathway in mediating the electrical stimulation induced muscle fibre transformation. And we found that the LFES program appears to particularly suited to counteract slow fibre reduction.

To summarize, the current study demonstrates that ES program is an efficient modality for improving slow fibre numbers (LFES) and fibre CSA (HFES). We also illustrates that a novel pathway regulates slow fibre expression and the role of electrical stimulation in ameliorating the slow muscle fibre reduction caused by chronic hypoxia-hypercapnia exposure. We found that LFES could effectively elevate slow fibre numbers both in SOL and GC through regulating the microRNA/Sox6 pathway. However, the HFES program could only activate this pathway in GC. This work provides new insights into understanding the role and the benefits of ES on muscle rehabilitation in COPD patients, and long-term use of the ES program in improving muscle function in COPD-induced muscle dysfunction.

## Methods

### Ethics statement

All animal handling procedures and experimental protocols were approved by the Ethics Committee of Wenzhou Medical University (wydw2015-0057). All experiments were performed in accordance with the relevant guidelines and regulations of the Laboratory Animal Unit of Wenzhou Medical University. All efforts were made to reduce the number of animals and to minimize their suffering.

### Animals

Fifty male Sprague-Dawley rats weighing 160 ± 21 g and aged 6 months were purchased from the Laboratory Animal Centre of Wenzhou Medical University. Rats were housed in the animal care facility with 12 h light/dark cycles and the house temperature was kept at approximately 23 °C. Food and water were available ad libitum.

### Study design

Rats were randomly divided into five groups: the normal control group (NC, n = 10), the hypoxia-hypercapnia+ sham stimulation group (HS, n = 10), the hypoxia-hypercapnia group (HH, n = 10), the hypoxia-hypercapnia+ low frequency electrical stimulation group (LFES, n = 10), and the hypoxia-hypercapnia+ high frequency electrical stimulation group (HFES, n = 10). The chronic intermittent hypoxia-hypercapnia model was established according to Xin-long Huo *et al.*^45^. Rats in the latter four groups were raised under a low O_2_ gas mixture (9–11% O_2_ and 5–6% CO_2_). The exposure time was 8 h/d, 7 d/w for 4 weeks. During the latter 2 weeks, the LFES group and the HFES group underwent the electrical stimulation protocols.

### Electrical stimulation protocols

The fur over the GC was carefully shaved; then, the HS, LFES, and HFES groups were properly pinioned, and 2 electrodes, measuring 9 cm^2^ (3*3 cm), were placed at the GC of both hind limbs of each rat. A portable battery-powered stimulator(HANS-200A, GENSUN MEDICAL, Nanjing, China) was used. During the training program, angular wave pulsed currents at 300 μs (pulse width) with a warm-up phase of 1 s, a stimulation phase of 2 s, and a fall time of 0.5 s was applied. The work-to-rest ratio of one on and one off was delivered in both stimulation interventions. The stimulation frequency was set to 30 Hz in the LFES group and 100 Hz in the HFES group. Either the LFES or the HFES group received one training program per day, and the period of each training program was 1 h. The total training program lasted 2 weeks (14 sessions). Stimulation intensity was strictly tested before the experiment and was judged by the reaction of the rats.

### H&E staining

Tissues were quickly removed after chloral hydrate injection. The GC and SOL were immediately fixed with a 4% paraformaldehyde solution and paraffin-embedded. The paraffin-embedded tissue block was sectioned at 3 μm thickness on the slicing machine (Leica, Germany). The sections were stained with haematoxylin-eosin (C0105, Beyontime, China) for the demonstration of inflammation and change in fibre cross-sectional area. The muscle fibre cross-sectional area was analysed using ImageJ software.

### Muscle fibre type identification

The slides were deparaffinised in 2 changes of xylene, 15 min each. The slides were rehydrated in 100% alcohol for 2 changes, 3 min each, and then transferred once through 95%, 70% and 50% alcohol for 3 min each. A ten min 3% H_2_O_2_ solution incubation was used to block endogenous peroxidase activity. Then, the slides were placed into 10 mM citrate buffer and high pressure antigen retrieval was carried out to unmask the antigenic epitope. The slides were blocked by 10% goat serum for 30 min and incubated with anti-slow myosin heavy chain antibody (1:4000, Ab11083, UK) at 4 °C overnight. The slides were washed in PBS 3 times, 5 min each. Secondary antibody incubation was undertaken according to the manufacturer’s instructions (PV-9002, ZSGB, China). The slides were washed in PBS and DAB solution was applied before counterstaining the slides with haematoxylin for 7 min.

### Immunofluorescent labelling of Sox6 and MHC-I

GC muscle was embedded with O.C.T. compound, frozen in liquid nitrogen-cooled isopentane, and cut into 8 mm thick cryosections with a cryostat (Thermo Scientific). The sections were blocked by 10% goat serum for 30 min and then incubated with primary antibodies against Sox6 (1:1000, Ab30455, UK) and MHC-I (1:2000, Ab11083, UK) at 4 °C overnight. After washing with PBS, secondary antibodies, including goat anti-rabbit (1:200, Dylight, USA) and goat anti-mouse (1:200, Dylight, USA), were used to conjugate primary antibodies for 1 h at room temperature. After a PBS wash, the slides were stained with DAPI (1:1000, C1005, Beyotime, China) and finally mounted with Antifade Mounting Medium (P0126, Beyotime, China). Pictures were captured by the Olympus FluoView FV500 confocal microscope.

### Western blotting

Equal amounts of protein (SOL: 20 μg, GC: 20 μg) were resolved in SDS-polyacrylamide gels and electrotransferred onto PVDF membranes (Millipore, USA). PVDF membranes were blocked for 2 h at room temperature with 5% non-fat dry milk. After washing in PBS, the membranes were incubated with primary antibodies against MHC-I (1:2000, Ab11083, UK), Sox6 (1:1000, Ab30455, UK), ERRγ (1:1000, Ab128930, UK), PPARβ (1:200, Santa Cruz H-74, USA), or tubulin (1:1000, Beyotime, China) at 4 °C overnight, and subsequently incubated with goat anti-rabbit (1:2000, Biosharp, China) or goat anti-mouse (1:2000, Biosharp, China) secondary antibodies at room temperature for 1 h. The immunoreactive bands were detected by Western Bright Sirius (Advansta, USA).

### RNA extraction and qRT-PCR analysis

Mir-499/208b transcript levels were measured using a Roche LightCycler 480 real-time PCR system (Roche Co., Germany) with the SYBR Green detection system. Total muscle DNA extraction was undertaken as follows. First, 150 mg GC and 150 mg SOL were extracted by 1 mL Trizol reagent (Invitrogen, USA). Then, 200 mL of a chloroform and isoamylol mixture (24:1) was added and mildly shaken for 30 s, followed by precipitation for 5 min and centrifugation for 10 min at 12,000 g, 4 °C. The supernatant was removed and washed with 70% alcohol twice, followed by centrifugation for 2 min and 5 min at 12000 g, 4 °C. Finally, the RNA was resolved with DEPC-H_2_O. The relative microRNA copy number was defined as the ratio of microRNA to U6. The primers for the mir-499 gene were mir-499-forward: 5′-GGGGTTAAGACTTGCAGTG-3′ and mir-499-reverse: 5′-CAGTGCGTGTCGTGGAGT-3′. The primers for the mir-208b gene were mir-208b-forward: 5′-GTGCAGGGTCCGAGGTATTC-3′ and mir-208b-reverse: 5′-AAGCTTTTTGCTCGCGTTATG-3′. The primers for the U6 were U6-forward: 5′- GCTTCGGCAGCACATATACTAAAAT-3′ and U6-reverse: 5′- CGCTTCACGAATTTGCGTGTCAT-3′. The primers for the MHC-I gene were MHC-I-forward: ACCCAGCAGAAGAAGACGTT and MHC-I-backward: TGCAGGTCACAATCACCACT. The primers for the MHC-IIa gene were MHC-IIa-forward: GTGCGTGAAGATGCTGTTGG and MHC-IIa-backward: CGTTTCCCGAGTCTCCTCAC. The primers for the MHC-IIx were MHC-IIx-forward: TCAGACCCACGGTCGAAGT and MHC-IIx-backward: CCACCACAAACACGGATGAC. Each real-time PCR reaction (25 μl total volume) contained cDNA (1 μl), 5×R-PCR Buffer (5.0 μl), 250 mM Mg^2+^ (0.3 μl), 10 mM dNTP (0.75 μl), 0.5 μl of each of the forward and reverse primers, 25×SYBR Green I (Bio-Rad, 1.0 μl), 10^−3^× Calibration (Bio-Rad, 1.0 μl), 5 U/μl HS-Ex-Taq (Takara, 0.25 μl), and ddH_2_O (14.7 μl). All data points were performed in triplicate.

### Statistical Analysis

The data were analysed by 1-way ANOVA followed by a post hoc comparison test using LSD (equal variances assumed) or Dunnett’s T3 (equal variances not assumed) method when more than 2 groups were compared. The data represent the mean ± SEM, with a statistically significant difference defined as *P < *0.05. Those statistical procedures were all performed with SPSS19.0.

## Additional Information

**How to cite this article**: Huang, S. *et al.* Electrical stimulation influences chronic intermittent hypoxia-hypercapnia induction of muscle fibre transformation by regulating the microRNA/Sox6 pathway. *Sci. Rep.* **6**, 26415; doi: 10.1038/srep26415 (2016).

## Supplementary Material

Supplementary Information
