# Supplementary material for: Electrical stimulation influences chronic intermittent hypoxia-hypercapnia induction of muscle fibre transformation by regulating the microRNA/Sox6 pathway
Source: Sci Rep. 2016 May 20;6:26415. doi: 10.1038/srep26415 (PMC4873781; doi:10.1038/srep26415)

Electrical stimulation influences chronic intermittent hypoxia-hypercapnia induction of muscle fibre transformation by regulating the microRNA/Sox6 pathway

Shiyuan Huang[1#], Lu Jin[1#], Jie Shen[1], Ping Shang[1], Xianxun Jiang[1] and Xiaotong Wang[1*].

1. The Centre of Neurology and Rehabilitation, the Second Affiliated Hospital of Wenzhou Medical University, Wenzhou, China.
*Corresponding author: Wangxt22@163.com
[#] These authors contributed equally to this work.

Corresponding author: Prof. Xiao-tong Wang. Tel.:+86 13706786183; Fax: +86 576 86666520.
E-mail address:wangxt22@163.com
Address: 109 Xueyuan Road, Wenzhou, Zhejiang; Postcode: 325027, Centre of neurology, the second affiliated hospital of Wenzhou Medical University, Wenzhou, China.

We use a single membrane instead of the entire membrane to cover each target protein respectively.

Here are the full-length gel images for Fig.2B, Fig.3A, Fig.4, Fig.6B, Fig.7A and Fig.8.

Fig.2B

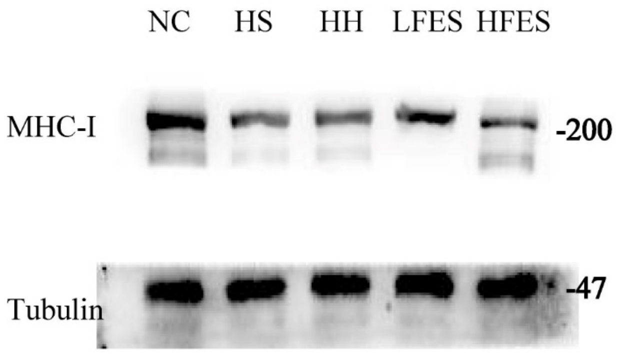

Fig.3A

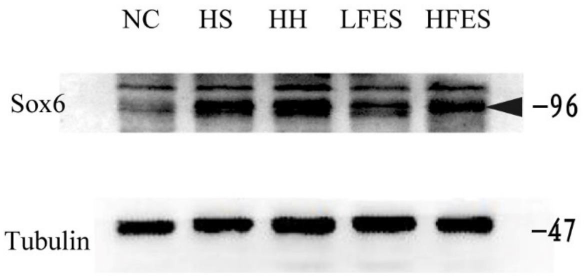

Fig.4

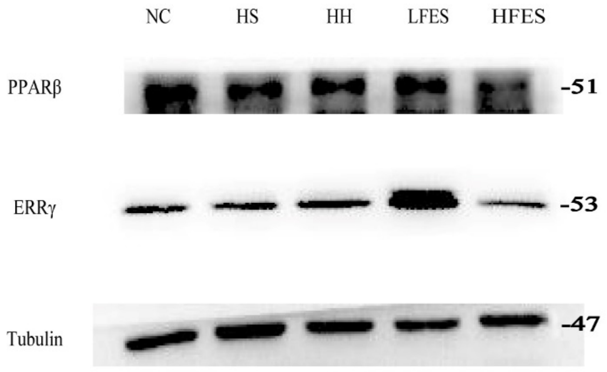

Fig.6B

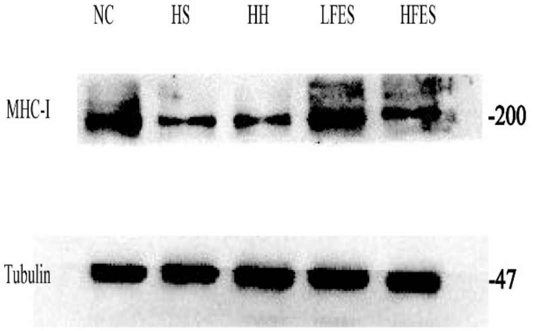

Fig.7A

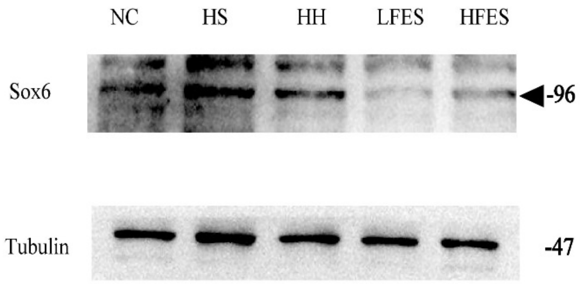

Fig.8

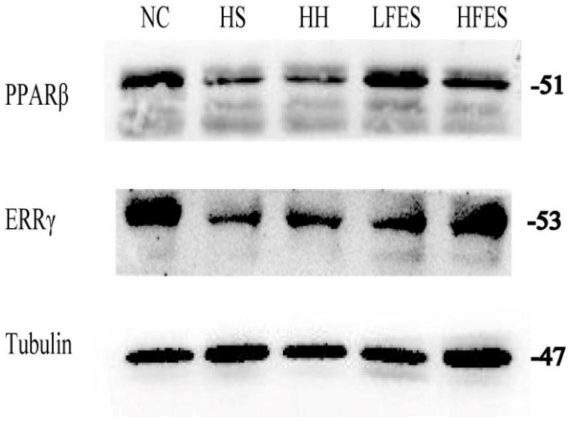

Supplement: Supplementary Information [file srep26415-s1.pdf]
